# Characterization of LaFeO_3_ Dielectric Ceramics Produced by Spark Plasma Sintering

**DOI:** 10.3390/ma17020287

**Published:** 2024-01-06

**Authors:** Pavel Ctibor, Josef Sedláček, Ksenia Illková, Libor Straka

**Affiliations:** 1 The Czech Academy of Sciences, Institute of Plasma Physics, Za Slovankou 3, 182 00 Prague, Czech Republic; illkova@ipp.asc.cz; 2 Faculty of Electrical Engineering, Czech Technical University, Technicka 2, 166 27 Prague, Czech Republic

**Keywords:** perovskite, LaFeO_3_, dielectrics, spark plasma sintering, photoconductivity

## Abstract

Commercially available LaFeO_3_ powder was processed using the spark plasma sintering (SPS) technique. The results of the dielectric measurement showed high permittivity, but this was strongly frequency-dependent and was also accompanied by a high loss tangent. The chemical purity of the powder and changes induced by the SPS process influenced the stability of the dielectric parameters of the bulk compacts. A microstructure with a homogeneous grain size and a certain porosity was produced. The microhardness of the sintered LaFeO_3_ was rather high, about 8.3 GPa. All the results are in reasonable agreement with the literature related to the production of LaFeO_3_ using different techniques. At frequencies as low as 100 Hz, the material behaved like a colossal permittivity ceramic, but this character was lost with the increasing frequency. On the other hand, it exhibited persistent DC photoconductivity after illumination with a standard bulb.

## 1. Introduction

Among the rare-earth orthoferrites with a perovskite crystal cell, LaFeO_3_ (labeled LFO) is known as an antiferromagnetic insulator. This ceramic exhibits an orthorhombically distorted perovskite structure that contains only trivalent iron atoms [1]. This means that the oxygen octahedra are tilted [2,3]. The chemical bond between Fe and O is mainly covalent, whereas the La-O bond was found to be ionic [4]. The LFO material belongs to the orthorhombic space group Pbnm with lattice parameters a = 5.556 Å; b = 5.565 Å; and c = 7.862 Å at room temperature. It has been shown that LFO also exhibits multiferroic behavior associated with the spontaneous lattice strain of about 2.4 × 10^−4^. An endothermic effect identified on calorimetric analysis, detected at about 1040 °C, represents an orthorhombic-to-rhombohedral first-order phase transition in LaFeO_3_ [5]. This transformation occurs without any mass change, which has also been confirmed by gravimetry.

LFO sintered using conventional approaches falls within the group of so-called colossal permittivity dielectrics (materials with values of ε_r_ over 10,000; in the case of LFO, this value is exactly 25,900 [6]). The loss tangent of LFO for the same samples is between 1.7 and 6.4, whereas the thermal coefficient of capacitance is high—about 30,000 ppm/K [6]. The electrical resistivity of LFO decreases with an increasing temperature [7,8], the cause of which is thermal activation of the charge carriers.

The observed high permittivity in this kind of ceramic is often also connected with its pore structure. As usual, all the four polarization mechanisms—electronic, ionic, orientation, and interfacial—contribute to the permittivity in the low-frequency range (under 10 kHz), whereas the interfacial polarization and also, to some extent, the orientational polarization are eliminated in the high-frequency range. The main problem of the already established LFO processing lays in its low reproducibility, which, therefore, results in a large variation in the electrical and multiferroic performance of the final product.

LFO is a very versatile material, with importance as a photocatalyst [9,10,11], an electroceramic in combination with the structurally similar material BaTiO_3_ [12], and a nanomaterial for sensors [13] or multiferroic material [14]. LFO has attracted the attention of researchers due to its structural aspects [15,16] and mechanical properties [11,17].

Based on our estimations, there exists room to improve the loss tangent and thermal coefficient of LFO capacitance via a proper method of sintering. The combined pressure-assisted and electrically driven process, i.e., spark plasma sintering (SPS), can produce very fine-grained materials if a correspondingly fine starting powder (e.g., nanometric) is utilized. The fast consolidation in SPS maintains the fine size of the powder and, correspondingly, the uniformity of the grain size distribution. For the majority of ceramics, SPS is also a very efficient way to reach proper densification of the material quickly. According to our knowledge, there is an absence of publications devoted to the testing of LaFeO_3_ dielectric ceramics produced using SPS. A brief summarization of our experience with this task is described below.

## 2. Experimental Data

### 2.1. Sample Manufacturing

**Figure 1:**
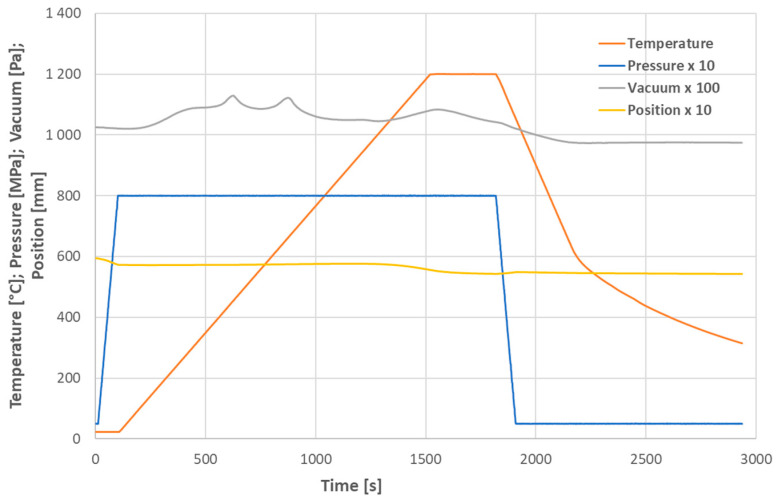
Time dependence of selected SPS parameters for sample SPS1200: temperature (°C), pressure (MPa), vacuum (Pa), position (mm). The scales are multiplied by factors of 10 or 100 as indicated.

A commercially available LaFeO_3_ powder (code LA-FEO-02; American Elements, Los Angeles, CA, USA) was processed in an SPS machine (10-4, Thermal Technology LLC, Minden, NV, USA). The carbon die was filled with the powder, and uniaxial pressure was applied from the top and bottom via carbon pistons. The key processing variables included the maximum temperature, mechanical pressure, stationary dwell time, and heating/cooling ramps. Cooling was performed at the maximum rate available for the SPS device, which is not equipped to cool the carbon parts directly, i.e., we used “free cooling”. This means that down to about 600 °C it was faster than heating, and below this temperature, it was slower than heating. The whole SPS cycle is displayed in Figure 1 and the parameters are listed in Table 1. The full compressive force was applied just before heating to compress the powder significantly. The produced samples were cylinders that were 16 mm in diameter and 4 to 5 mm in height. Because of the highly critical fragility of the sintered samples, of the three tested temperatures, i.e., 1150, 1200, and 1300 °C, only the sample SPS1150 was able to survive the complete dielectric testing (clamping in the fixture and thermal drift) (Table 1). The other samples broke and were suitable only for some supporting tests, such as X-ray diffraction (XRD) or dilatometry.

### 2.2. Microstructure Characterization

A polished cross-section was prepared from the SPS-processed LFO. The sample was sectioned using a diamond cutting saw and mounted in a low-viscosity polymeric resin. Polishing was carried out using the automatic machine Tegramin-25 (Struers, Copenhagen, Denmark). The cross-sectional view was observed via scanning electron microscopy (SEM) using a Phenom-Pro microscope (Thermo Fisher Scientific, Breda, The Netherlands). This apparatus is equipped with a CeB_6_ thermionic cathode, and the LFO samples were observed using the backscattered electron (BSE) mode. The images were collected at a 15 kV electron-beam voltage.

Vickers microhardness of the LFO was measured on polished cross-sections using an optical microscope with a Hanemann head holding a Vickers indenter. A working load of 1 N was applied for 15 s. The mean microhardness was calculated as an average value from 20 indentations.

Phase composition was evaluated by means of X-ray diffraction (XRD), carried out using the powder diffractometer D8 Discover (Bruker, Mannheim, Germany). Bragg–Brentano geometry with a 1D detector and Cu-Kα radiation was used. The scanned region started from 20 to 110° 2θ with a 0.03° 2θ step size and a counting time of 192 s per step. The obtained diffraction patterns were subjected to a software treatment (Bruker AXS DIFFRAC.TOPAS 5).

### 2.3. Thermal Characterizations

Thermal behavior was analyzed using a Setaram Themys instrument (Setaram, Caluire-et-Cuire, France). Differential thermal analysis (DTA) was carried out at a heating rate of 10 °C/min from 50 °C to 1500 °C in an Ar atmosphere with a flow rate of 5 L/h. The analyses were performed on about 30–50 mg of LaFeO_3_ powder with an empty reference crucible. Before the analysis, the sintered sample was ground in a mortar.

Thermal expansion was studied using a vertical pushrod dilatometer, TMA Setsys 16/18 (Setaram, Caluire-et-Cuire, France). Measurement was performed on a free-standing sintered sample 4 mm long in a controlled Ar flow. A heating rate of 10 °C/min was used in the temperature range from 30 °C to 1500 °C. A load of 5 g was applied. The obtained data were corrected via blank subtraction.

### 2.4. Dielectric Parameters

Dielectric measurement of the LFO cylinders was carried out. The faces of the sample were plated with aluminum electrodes in an evaporating apparatus. Before this metallization, LFO was ground using SiC paper to eliminate differences in uneven surfaces. Using a mask, a three-electrode system was used to diminish the stray current influence during the measurement. One face was Al-coated completely and the second one had an internal working electrode, 12 mm in diameter, and an external ring electrode, earth-connected during the measurements.

An electric field was applied in the same direction as the mechanical compression at the SPS (i.e., perpendicular to the cylinder face). Dielectric capacitance of the samples was measured using a programmable impedance analyzer model 4284A (Agilent, Santa Clara, CA, USA) and a high-precision sample fixture 16451B (Agilent).

The dielectric constant (i.e., relative permittivity) was calculated according to the following equation (Equation (1)):

ε_r_ = C × d/ε_0_ × A (1)

where ε_r_ represents the relative permittivity, C (F) means the equivalent parallel capacitance, d (m) is the thickness of the sintered LFO, A (m^2^) is the area of the working electrode, and ε_0_ is the vacuum permittivity (8.85 × 10^−12^ F/m). Measurements at elevated temperatures were carried out using a programmable furnace Novotherm (Novocontrol, Montabaur, Germany) equipped with a special Broadband Dielectric Spectroscopy (BDS) sample fixture 1200 (Novocontrol, Montabaur, Germany).

When a sinusoidal alternating voltage is applied to an ideal capacitor, the current advances by π/2 in phase. In the case of a practical capacitor, however, the advance in phase is (π/2 − δ), which is smaller than π/2. Angle δ is referred to as the loss angle. The loss tangent (or “dissipation factor”) is numerically equal to the ratio of the active dissipative current to the reactive (charging) current (Equation (2)).

tan δ = cot θ = 1/(2π × f × Rp × Cp)(2)

where δ is the loss angle, θ is the phase angle, f is the frequency, R_p_ is the equivalent parallel resistance, and C_p_ is the equivalent parallel capacitance. In the BDS measurement system, tan δ was recorded automatically with the corresponding capacitance. During the elevated temperature measurement, other parameters like AC conductance and complex impedance were recorded as well.

Electric resistance was examined at room temperature with a resistometer—Keithley, model 6105. The DC electric field was applied from a regulated high-voltage source and the values were recorded with an electrometer (617C, Keithley Instruments, Solon, OH, USA). The magnitude of the applied voltage was 100 ± 0.05 V. The volume resistivity calculation was based on the measured resistance and the specimen dimensions.

## 3. Results and Discussion

### 3.1. Powder Thermal Stability and Chemical Purity

**Figure 2:**
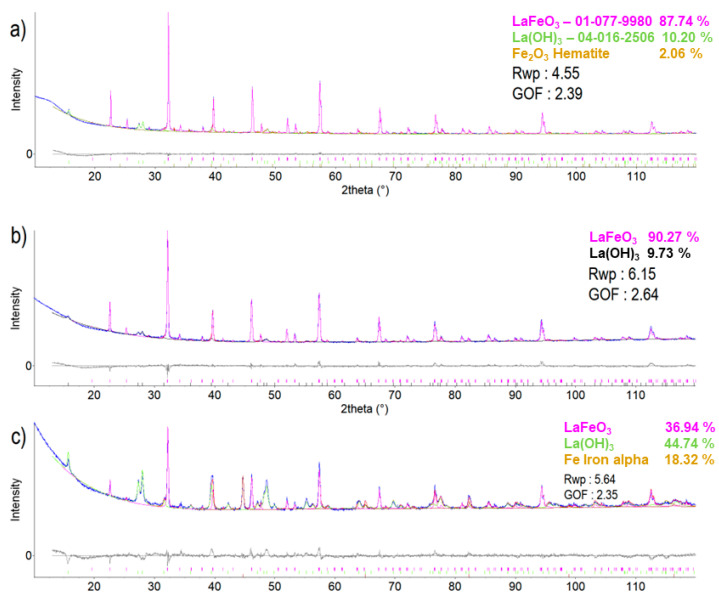
XRD patterns of the samples—from the top: (**a**) powder, (**b**) SPS1150, (**c**) SPS1300.

Unfortunately, the commercial powder was hydrated. Our XRD analysis showed over 10 percent of La(OH)_3_ in its composition, and, moreover, approximately 2 percent of hematite Fe_2_O_3_; Table 2 and Figure 2. Processing in SPS at a low temperature, 1150 °C, led to the disappearance of hematite and a nearly complete preservation of other components. However, when sintering was performed at a high temperature, 1300 °C, the enhanced formation of La(OH)_3_ and also of metallic iron was detected. Hydrogen came most probably from the ambient moisture. The preservation of lanthanum hydroxide from the starting powder in sample SPS1150 seems realistic. Most probably, after thermal volatilization of Fe from LaFeO_3_, the formed La_2_O_3_ was prone to hydration directly after cooling according to the reaction La_2_O_3_ + 3 H_2_O → 2 La(OH)_3_.

**Figure 3:**
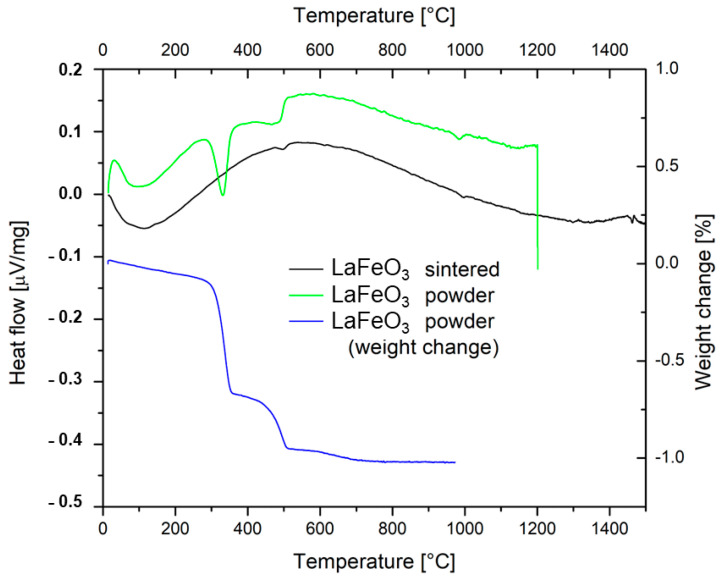
Thermal analyses of the LFO powder and the sintered sample.

The DSC heating curve showed three endothermic effects with the peak maximum temperatures of 325 °C, 480 °C, and 980 °C, respectively; Figure 3. The first endothermic effect is associated with dehydration of the powder (moisture removal from the pores). Since lanthanum oxide is easily converted to the hydroxide form, the endothermic peak at 480 °C corresponds to the dehydration of lanthanum hydroxide present in the powder (cf. Table 2) at heating. A repeated test with the same sample showed no more peaks up to 950 °C. Both processes together were accompanied by a mass loss of approximately 1%, visible on the TGA curve, and, after them, the sample mass became stable. Above 700 °C, the TGA curve did not show any mass change. The third, smaller, endothermic effect on the DSC curve at about 980 °C could correspond to the orthorhombic-to-rhombohedral first-order phase transition, as mentioned in the literature [5], or to melting. According to the supplier information, the melting temperature T_m_ of the LFO powder is 977 °C, but a clear melting peak was not observed.

The Archimedean density of the sample SPS1150 was 96.2% of the crystallographic density (CD). The CD was considered (6.637 g/cm^3^ [6]) and the measured Archimedean density was 6.386 g/cm^3^. For a comparison, 94.6% of CD was obtained for LFO processed via conventional sintering [6].

**Figure 4:**
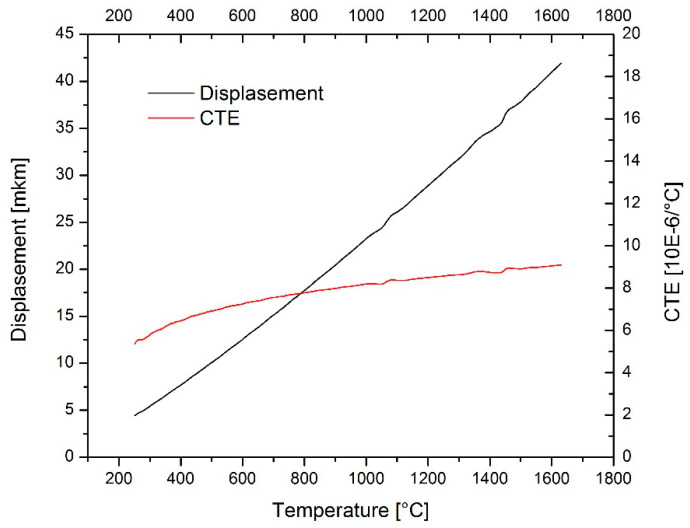
Thermal expansion of the sintered LFO (displacement shown in µm).

Since the thermal expansion continued well behind 977 °C, Figure 4, we could exclude melting at this temperature. The sintered LFO sample remained solid to at least 1600 °C. To recognize a true melting point of the material was not our goal, besides other reason for a limited instrumental range. When we consider the sintering temperature as T_s_ = 0.7 × T_m_ (T_m_ is the melting point, and T_m_ is 1600 °C or higher, for a simplicity we consider 1600 °C), we obtain Ts = 1120 °C. All applied SPS temperatures in Chapter II were above this temperature, and in this way proper sintering was ensured. The value of the coefficient of thermal expansion (CTE) increased with temperature and reached its maximum at about 1600 °C with 8.5 × 10^−6^/°C. In the literature, only the low-temperature CTE was found, 6 × 10^−6^/°C (for 327 °C) [18], similar to the value of our LFO at the same temperature.

### 3.2. Dielectric Characterization

**Figure 5:**
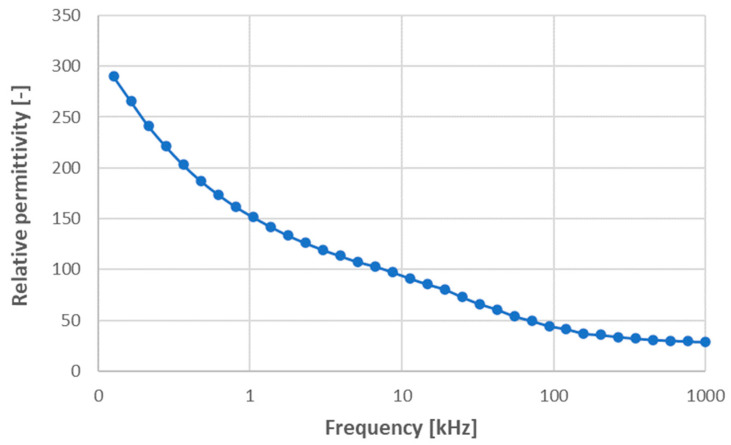
Relative permittivity versus variable frequency, sample SPS1150.

Relative permittivity corresponding to room temperature is displayed in Figure 5. The values are fairly high, but the slope of the curve is extremely large as well. These results were consistent with the knowledge that the charge polarization (or relaxation polarization) of structurally dense materials with appreciable mobility of charge carriers produces considerably larger relative permittivity at low frequencies [6,12].

**Figure 6:**
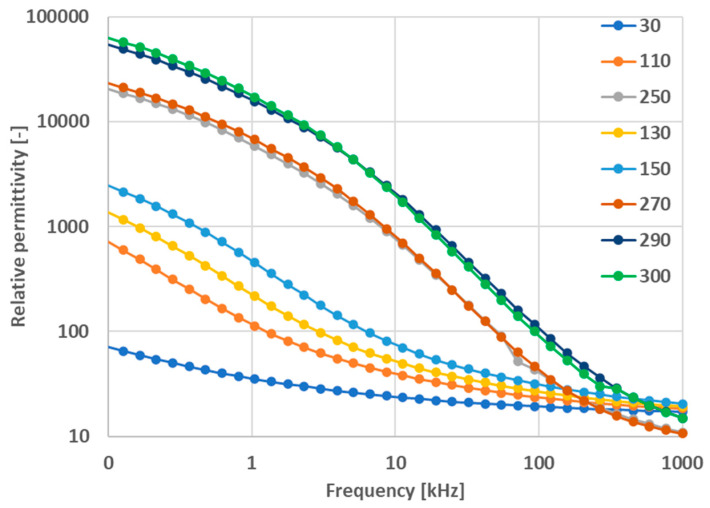
Relative permittivity versus variable frequency for various temperatures, sample SPS1150.

The permittivity curves measured at elevated temperatures up to 300 °C are shown in Figure 6. We could recognize three temperature regions with different dielectric behaviors. The first region is the room temperature 30 °C, where the maximum permittivity is under 100. (Please note that, because of a different sample fixture, there is no perfect alignment of the “room temperature measurement”, Figure 5, and “the elevated temperature measurement” without actual elevation, Figure 6, curve 30). The second region is between 100 °C and 150 °C, where the maximum permittivity lies between 900 and 3000. The third region is represented by temperatures above 250 °C with over 20,000 as the maximum permittivity value. There is a jump between 270 °C and 290 °C, where, for the temperature difference of 20 °C, the permittivity value is more than double. The frequency dependence is stronger at elevated temperatures, where, besides the true polarization (space charge polarization in this frequency region), the conduction is also thermally activated. Possible interfacial effects between the electrodes and dielectric material are considered as space charge polarization in the present case. Up to 300 °C (i.e., the maximum measurement temperature), there is no reason for instability in aluminum metallization (i.e., the embedded electrodes).

**Figure 7:**
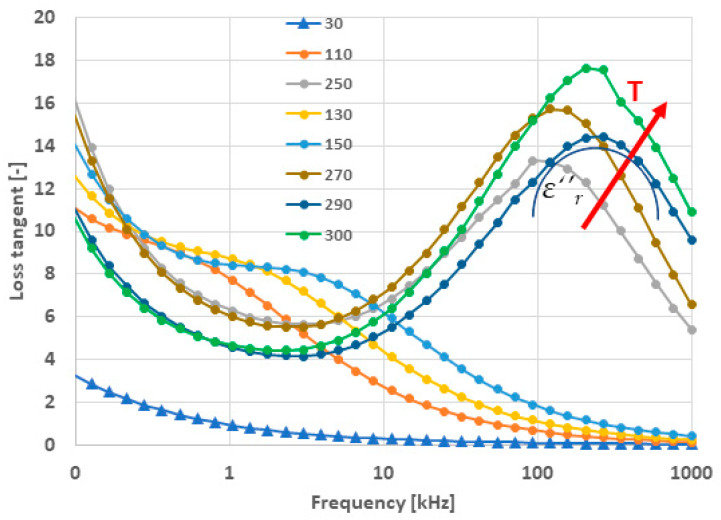
Loss tangent versus variable frequency, sample SPS1150. Temperatures up to 300 °C are shown.

Loss tangent curves are shown in Figure 7. The run of the elevated temperature loss tangent curves is comparable with the literature [6,7,8,9,19]. The existence of two separable components of losses could be recognized [9]. The high-frequency component is one, where *ε*″*_r_* corresponds to the part of the losses associated with the relaxation phenomena (cf. the semicircular halo in Figure 7 with maxima above 100 kHz). The low-frequency component exists due to a purely conductive mechanism.

For the measurement at 150 °C, there is a small peak placed around the 5 kHz frequency. The same phenomenon was observed by another research team [20] and associated with a Maxwell–Wagner (M–W) mechanism. According to this mechanism, the material consists of a highly resistive grain boundary and less-resistive grain (resulting in an internal barrier layer capacitor) and yields a giant permittivity.

We could say that because of impurities in the powder, which are not entirely eliminated via sintering, the conductive component dominated at very low frequencies. The redistribution of resistivity between grains and grain boundaries (M–W mechanism) took place at medium frequencies. And, finally, the relaxation of permittivity was the governing factor at high frequencies, like in all high-permittivity materials. However, similarly to CCTO ceramics [21], the LFO application prospective would mainly involve a material preserving its colossal permittivity up to at least 50 kHz, which is not the case of actual SPS-processed LFO.

The temperature coefficient of capacitance (TCC) was calculated [22] for the interval between 30 °C and 130 °C. Its value was +1260 ppm/K for the sample SPS1150. This value is significantly lower than in the literature [6]. A low TCC value is desirable to ensure stability of the dielectric behavior of a circuit part during unavoidable in-service thermal fluctuations. The combination of room-temperature dielectric properties obtained at 1 MHz, i.e., relative permittivity 17, loss tangent 0.05, and temperature coefficient of capacitance +1260 ppm/K, makes the SPS-processed LaFeO_3_ a material with large thermal sensitivity to the dielectric properties. Spark plasma sintering of LFO perovskite promoted increased dielectric and capacitive proprieties, unfortunately with an extinction of the colossal value of *ε*’ at about 1 kHz and at the highest studied temperatures (approaching 300 °C). In industrial applications in the capacitor branch, we cannot admit the TCC at 1 MHz as high as +1260 ppm/K. Different is the branch of sensors, where a pronounced response of a simply measurable parameter (here we discuss capacitance; elsewhere, resistance [7] is reported) to an external factor is advantageous.

The volume DC resistivity of the representative sample, i.e., SPS1150, was 68,950 Ωm. LaFeO_3_ is an n-type semiconductor with electrons as the majority charge carriers [9]. In contrast, screen-printed LaFeO_3_ thick film had the characteristics [7] of a p-type semiconductor (holes as the majority carrier). This should depend also on the production history of the sample, since the thermoelectric calculations confirmed that LaFeO_3_ prefers holes as the charge carriers, with a p-type character up to 75 GPa of mechanical pressure, and then switches to electron carriers under 100 GPa [23]. Generally, LFO belongs to mixed ionic and electronic conductors (MIECs) [24]. Because of oxygen vacancies, the reported impedance of bulk LaFeO_3_ was in the order of 10^3^ Ω [25], whereas in our case is it about 10^6^ Ω.

**Figure 8:**
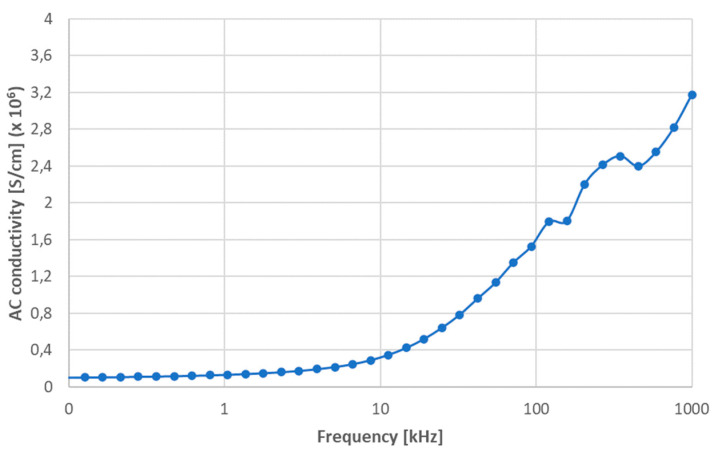
Real part of AC conductivity of the sample SPS1150 measured at room temperature.

The dependence of electrical AC conductivity, more exactly its real part (σ’_ac_), on a varying frequency is displayed in Figure 8, as measured at room temperature. A relationship between frequency and conduction can be observed in the figure. At low frequencies, σ’_ac_ exhibited a nearly flat response; however, at higher frequencies (more than 10 kHz), σ’_ac_ began to increase with frequency, and at extremely high frequencies its growth started to be partly instable. The occurrence of space charges, as well as cationic disarray between neighboring sites (cf. the combination of covalent and ionic bonds, mentioned in Introduction), could explain why σ’_ac_ increased so steadily with the frequency [26].

**Figure 9:**
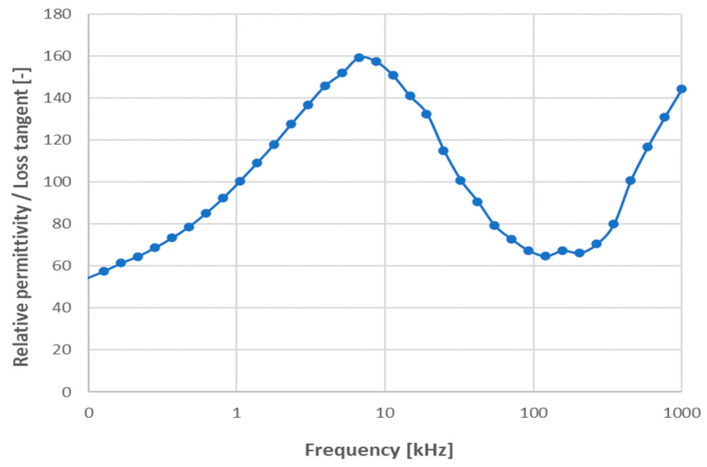
Ratio *ε*’/*tan δ* against frequency for the sample SPS1150, at room temperature, displayed versus the changing frequency.

**Figure 10:**
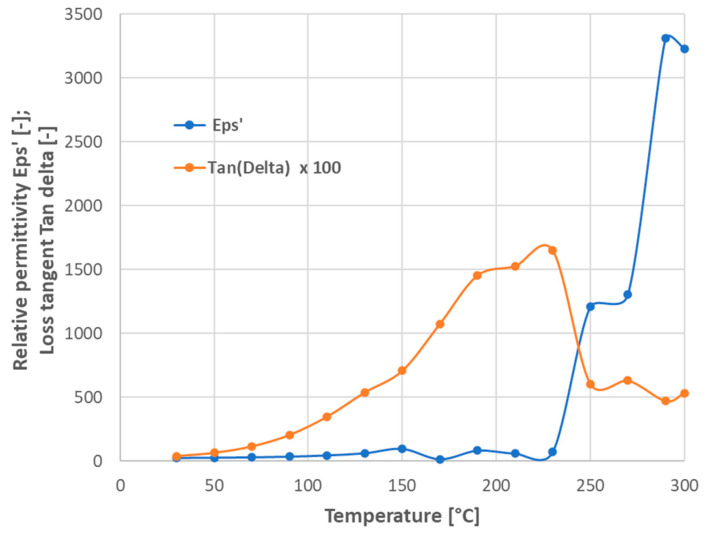
Permittivity and loss tangent of the sample SPS1150 at a fixed frequency, 66,400 Hz, displayed versus the changing temperature.

High electronic conductivity and dielectric loss make the capacitive application of this LFO limited, but the high permittivity over a certain frequency range is its notable property, and an analysis was carried out to determine the best working conditions for this sample. To identify the frequency at which, proportionally, the highest *ε*′ and the smallest *tan δ* occur, the ratio *ε*’/*tan δ* against *f* was calculated. Figure 9 shows the factor obtained when *ε*’ was divided by *tan δ* (at 30 °C and at the frequency from 20 Hz to 1 MHz, for the SPS1150 sample). The maximum was identified at *f* = 66,400 Hz, which is the frequency that presents the best association of permittivity and dielectric loss for this sample (*ε*’ = 103 and *tan δ* = 0.649, respectively). At the fixed frequency of 66,400 Hz, variation in *ε*’ and *tan δ* with *T* for LFO was analyzed from 30 °C to 300 °C. The results are shown in Figure 10, where the maximum permittivity occurs at *T* = 290 °C, with *ε*’ = 3310. However, the dielectric loss increased with temperature and then decreased again.

### 3.3. Microstructure and Hardness

**Figure 11:**
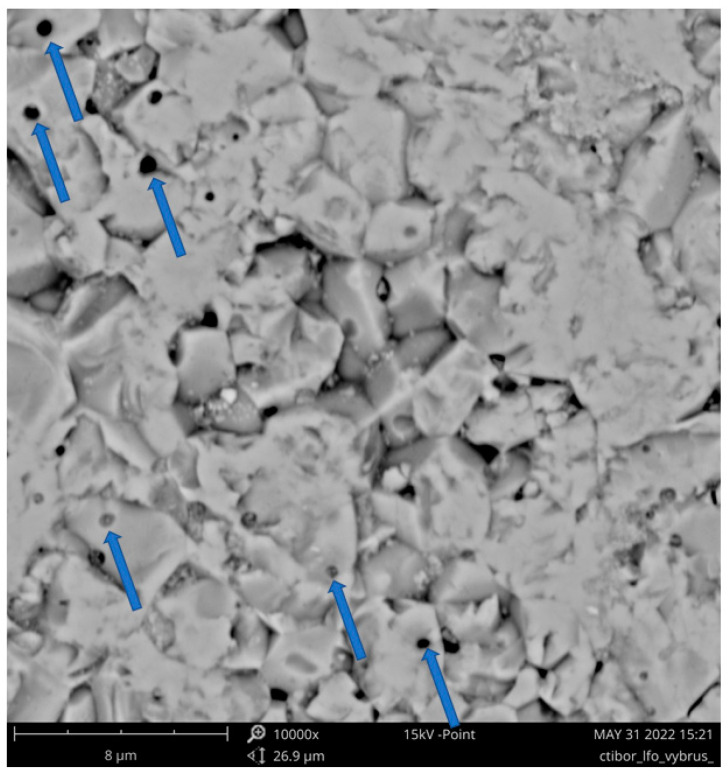
SEM micrograph of the LFO cross-section, sample SPS1150. Globular pores indicated by arrows.

A SEM micrograph of the sample, previously tested as a dielectric, shows grains with a size typically between 2 and 5 µm, Figure 11 (about two times finer than a furnace-sintered sample with 6h dwell at 1300 °C [6], but about two times coarser than a furnace-sintered sample with 1h dwell at 1300 °C [22]). Besides the macroscopic porosity, internal globular pores within individual grains were also rather frequent. These are indicated by the arrows in Figure 11, and their typical diameter was only about 300 nm to 500 nm.

The microhardness reached the value of 8.3 ± 1.6 GPa. A value of only 4 GPa [27] was reported for an LFO coating deposited through a molten carbonate surface conversion coating process. Rather expectedly, the bulk material is harder than the coating.

### 3.4. Electrical Response to Visible Light

**Figure 12:**
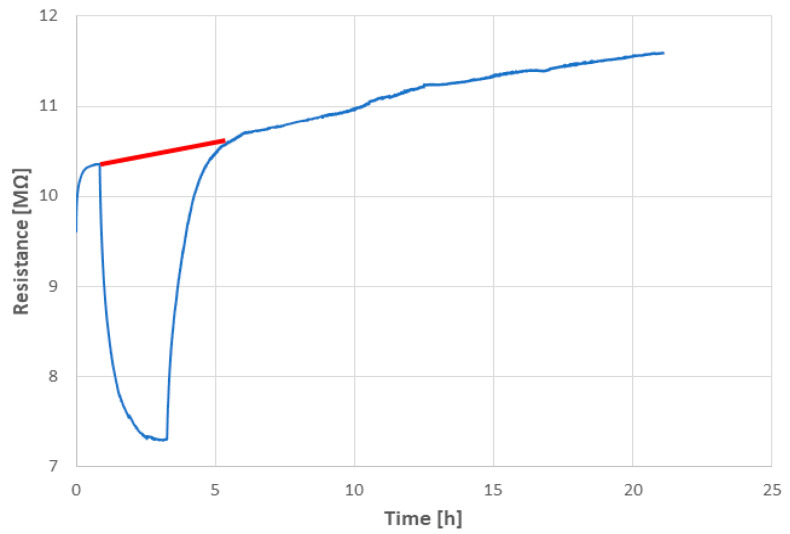
Photoconductivity plot of the sample SPS1150. The trend under dark conditions is shown with the red line.

The photoconductivity of the sample SPS1150 was tested using a standard 60 W bulb during the simultaneous measurement of electrical DC resistance with the approach described in the Experimental section. A light power of 20 mW/cm^2^ was used and the DC bias was set to 50 V. The run of the experiment is displayed in Figure 12.

The DC resistance run at the “dark conditions” was recognized to have an inherent monotonous increase in time, cf. the range between 10 and 20 h in Figure 12. The light was switched on after 0.85 h and switched off after 3.25 h from the beginning of the experiment. We saw a rather fast decrease in resistance under illumination, which was later saturated. This corresponds to the activation of charge carriers and their subsequent depletion. Photoconductivity at room temperature could be mostly ascribed to the increment in carrier density under illumination. The new “stable” value induced by light was at about 7.3 MΩ, i.e., a decrease to about 70% of the initial value (i.e., the value just before the enlightenment was considered 100%). After switching the light off, the recovery started. This process was, in our experiment, similarly fast as the decay (approximately 2.5 h). We can call this interval a persistent photoconductivity period. The initial linear trend, indicated in the graph with a red line, was further followed. The experiment showed a relatively large, but slow, fully recoverable, photo-induced change in resistance (or, inversely, also conductance). In principle, similar transient photocurrent responses were detected at electrochemical measurements by other authors [9]. A high and persistent photoconductivity at the oxide heterostructure of two insulators, LaFeO_3_ and SrTiO_3_, was described [28], but for a system consisting of a thin LFO film grown on a SrTiO_3_ single crystal. This arrangement is not relevant to the photoconductive response of the pure and self-supporting LFO, i.e., our case.

LFO samples could be excited by light with a wavelength equal to or smaller than 550 nm [11]. LFO in the form of nanometric powder was reported as an efficient photocatalyst [10]. Pure LaFeO_3_ nanoparticles showed good photocatalytic activity, and over 83% of methyl orange reagent was decomposed after 180 min of visible-light radiation, whereas UV-light is typically necessary for a similar effect to be gained with different photocatalytic substances. In the ABO_3_ perovskite cathode materials of fuel cells, B site cations are catalytically more active than A site cations for oxygen reduction reactions [29], i.e., in the LFO case, the Fe ions are more active than the La ions.

**Figure 13:**
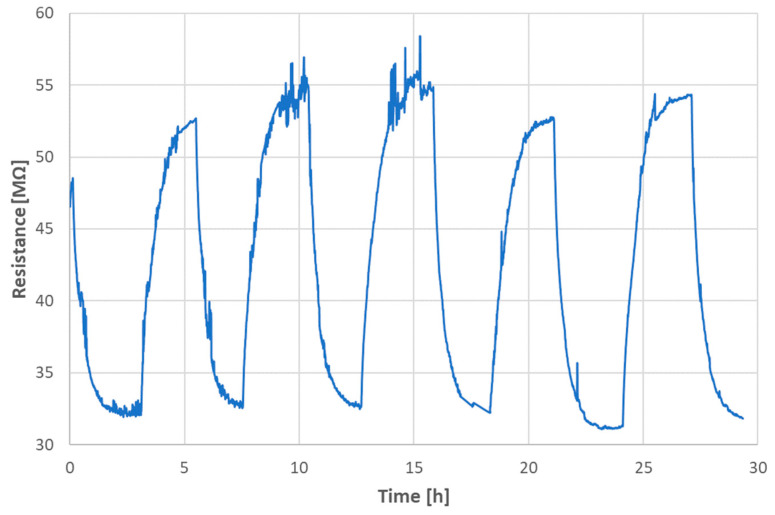
Reproducibility of the photoconduction phenomenon, sample SPS1150.

The reproducibility of the photoconduction phenomenon was monitored, Figure 13, for a total duration of 30 h. The “light-on” periods were characterized by the resistance mean value (3.20 ± 0.70) × 10^7^ Ω, and the “light-off” periods by the resistance mean value (5.38 ± 1.20) × 10^7^ Ω. The narrow “secondary” peaks, present because of fluctuations (various source instabilities and ripples), were abandoned for the calculations. The inherent increase in time, cf. Figure 12, was not confirmed for this longer period, Figure 13. The understanding of the stationary value of resistance of SPS-LFO must be improved with a next study.

**Figure 14:**
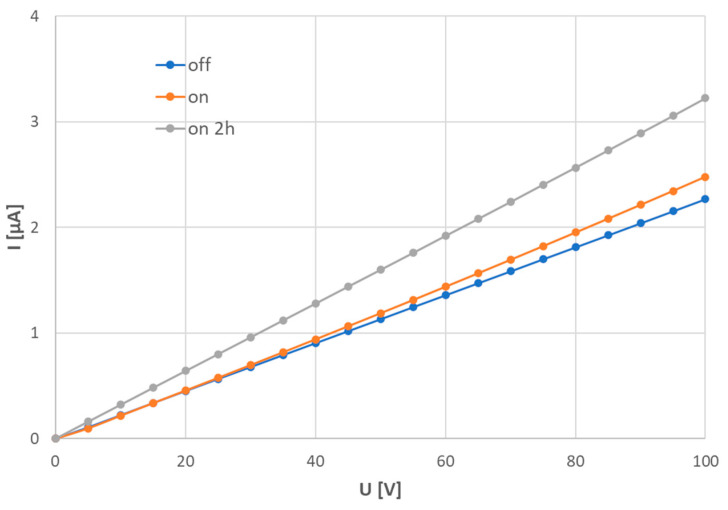
Volt-ampere characteristics of the photoconduction phenomenon, sample SPS1150.

A graph showing the volt-ampere characteristics (VACs), Figure 14, was produced using a sweep from 0 V to 100 V DC with a step of 5 V and a time step of 10 s. The current is shown in microamperes. Three linear dependencies are shown—VACs for the dark conditions (label “off”), VACs recorded immediately after turning the light on (label “on”), and finally VACs recorded on a sample conditioned under the light for 2 h (label “on 2 h”). The graph shows that the stronger the voltage, the more pronounced the light-induced photoconductance is, and the phenomenon is strictly linear. This would be perfect for application of the LFO sample in sensors.

Based on this subchapter, the most promising field for the application of an SPS-processed LFO is in the field of radiation sensors. Generally speaking, our SPS approach was able to produce dense and fine-grained ceramics, but it was not able to fully overcome the problems associated with the limited purity of the starting materials. The vague definition of the melting point of LFO made the setting of best possible SPS parameters also partly doubtful.

## 4. Conclusions

A commercial powder with a nominal LaFeO_3_ composition (LFO) was consolidated into compacts using the spark plasma sintering technique. Their microstructure exhibited homogeneous grain size and certain intragrain porosity. The Vickers microhardness of the LFO was rather high at slightly over 8 GPa. This factor was, however, associated with marked fragility, and many samples tended to crack during normal manipulation (e.g., surface polishing, dielectric fixture clamping).

The dielectric measurements showed high permittivity with a maximum of over 50,000, but this was highly frequency- and temperature-dependent, and was also associated with high loss tangent (over 15) at a frequency of 100 Hz. Losses also had a secondary maximum at about 0.5 MHz. The room-temperature dielectric parameters tended to stabilize in the radio-frequency range. At 1 MHz, their values were as follows: relative permittivity 17 and loss tangent 0.05. A temperature coefficient of capacitance of +1260 ppm/K was calculated for the temperature interval between 30 °C and 130 °C. The overall character of the sample generally agreed with the literature, i.e., extraordinarily high permittivity was detected only at a low frequency together with an elevated temperature. Other combinations of parameters led to the LFO ceramics having a more lossy character. In summary, various conventionally sintered LaFeO_3_ dielectrics were slightly overperformed with our SPS ceramics. However, this was valid only at a certain range of conditions. The colossal dielectric behavior of our SPS-LFO showed extinction at about 1 kHz. LFO exhibited persistent photoconductivity after illumination with a standard bulb, whereas the extent of the induced change was about 30% of the initial value. An increment in carrier density under illumination was responsible for this effect.

## Figures and Tables

**Table 1:** Processing parameters of the spark plasma sintering.

Sample	Ramp Up (°C/min)	Pressure (MPa)	Dwell at T_max_ (min)	Ramp Down (°C/min)
SPS1150	50	80	5	Free; details in text
SPS1200 *	50	80	5; 10	Free; details in text
SPS1300 *	50	80	5; 10	Free; details in text

Samples are labeled according to the T_max_ (°C). * Broken upon removal from the SPS die or during the necessary handling for dielectric tests.

**Table 2:** Phase composition according to XRD measurement, Rietveld-based quantification (wt.%).

Sample	LaFeO_3_	La(OH)_3_	Fe_2_O_3_	Fe
Powder	87.74	10.20	2.06	0
SPS1150	90.27	9.73	0	0
SPS1300	36.94	44.74	0	18.32

## Data Availability

The data serving for elaboration of this manuscript are non-public. They are stored by the authors and will be available to the readers upon email request, ctibor@ipp.cas.cz.
